# AI-assisted longitudinal cardiac phenotyping identifies domain-specific remodeling patterns in Fabry cardiomyopathy

**DOI:** 10.3389/frai.2026.1800355

**Published:** 2026-09-10

**Authors:** Kuo-Tzu Sung, Wen-Chung Yu, Ming-En Liu, Po-Lin Lin, Yau-Huei Lai, Hsiang-Wei Yang, Sheng-Hsiung Chang, Chung-Lieh Hung, Dau-Ming Niu

**Affiliations:** 1 Institute of Clinical Medicine, National Yang Ming Chiao Tung University, Taipei, Taiwan; 2 Division of Cardiology, Department of Internal Medicine, Taipei MacKay Memorial Hospital, Taipei, Taiwan; 3 Department of Medicine, MacKay Medical University, New Taipei City, Taiwan; 4 Division of Cardiology, Department of Medicine, National Taipei Veterans General Hospital, Taipei, Taiwan; 5 School of Medicine, National Yang Ming Chiao Tung University, Taipei, Taiwan; 6 Division of Cardiology, Department of Internal Medicine, Hsinchu MacKay Memorial Hospital, Hsinchu, Taiwan; 7 Mackay Junior College of Medicine, Nursing, and Management, New Taipei City, Taiwan; 8 Institute of Biomedical Sciences, MacKay Medical University, New Taipei City, Taiwan; 9 Department of Pediatrics, National Taipei Veterans General Hospital, Taipei, Taiwan; 10 Genetic Consultant Center Rare Disease Medical Research Center, National Taipei Veterans General Hospital, Taipei, Taiwan

**Keywords:** artificial intelligence, enzyme replacement therapy, Fabry cardiomyopathy, global longitudinal strain, longitudinal phenotyping, multidomain remodeling, pattern recognition, QRS duration

## Abstract

**Background:**

Cardiac involvement in Fabry disease spans structural hypertrophy, mechanical dysfunction, and electrical conduction abnormalities that rarely progress in parallel. Although such discordant longitudinal behavior is commonly encountered in clinical practice, it has not been systematically organized within a coherent longitudinal framework.

**Methods:**

We conducted a retrospective multicenter longitudinal study of 38 patients managed across three geographically distinct affiliated hospitals within the MacKay Memorial Hospital system, located in Taipei, Tamsui, and Hsinchu. Three major domain-specific remodeling patterns, including structural, mechanical, and electrical domains, were assessed and indexed by left ventricular mass index (LVMi), left ventricular global longitudinal strain (LVGLS), and QRS duration using standard 12-lead electrocardiography (ECG), respectively. All echocardiographic domain measures were conducted using an AI-assisted deep learning–based platform (Us2.ai), which enables standardized longitudinal processing of multi-parametric measurements. Domain-specific longitudinal within-patient changes were classified by the sign of the numerical change (positive vs. negative), with adverse or non-adverse designation defined according to the domain-specific clinical meaning of each parameter, and compared between patients with and without enzyme replacement therapy (ERT).

**Results:**

During a median of 5.3 years (interquartile range, 2.8–7.1 years) follow-up, electrical remodeling showed an adverse trend, with QRS duration increasing from 128.21 ± 31.18 ms to 139.63 ± 37.95 ms (Δ + 11.42 ms, 95% CI 5.93–16.92; *p* < 0.001). Mechanical remodeling assessed by LVGLS changed from −14.74 ± 5.83% to −16.69 ± 4.44% (Δ − 1.95 percentage points; 95% CI, −4.05 to 0.14; *p* = 0.067), whereas structural remodeling by LVMi showed no significant longitudinal change (*p* > 0.05). In the LVGLS analysis, concordant adverse remodeling was most frequent (18/38, 47.4%), followed by electrical-leading adverse remodeling (12/38, 31.6%), mechanical-leading adverse remodeling (5/38, 13.2%), and concordant favorable remodeling (3/38, 7.9%). The phenotype distribution differed significantly between untreated and ERT-treated patients (*p* < 0.001).

**Conclusion:**

AI-assisted longitudinal monitoring enables consistent characterization of non-parallel, domain-specific change patterns. Electrical–mechanical phenotypes were heterogeneous, with a significant difference in phenotype distribution between untreated and ERT-treated patients.

## Highlights

- AI-assisted analysis standardized serial echocardiographic measurements across baseline and follow-up examinations.
- Electrical, mechanical, and structural cardiac measures frequently changed in non-parallel directions within individual patients.
- Paired changes in QRS duration and LVGLS identified four clinically interpretable electrical–mechanical remodeling patterns.
- Phenotype distributions differed significantly between untreated and ERT-treated patients.
- Multidomain surveillance may identify clinically relevant change that is not apparent from a single cardiac parameter.

## Introduction

Fabry disease is an X-linked lysosomal storage disorder caused by deficiency of α-galactosidase A (Germain, 2010), leading to progressive glycosphingolipid accumulation across multiple organ systems, with cardiac involvement representing a central feature of adult disease (Linhart et al., 2020; Ortiz et al., 2018). At the myocardial level, storage is not confined to a single compartment but affects cardiomyocytes, the cardiac conduction system, and the myocardial interstitium and microarchitecture (Pieroni et al., 2021), resulting in a spectrum of abnormalities spanning structural remodeling, mechanical dysfunction, and electrical conduction disturbances (Vijapurapu et al., 2019a). Prior studies have consistently shown that left ventricular hypertrophy (LVH) and mass accumulation, impaired myocardial deformation (Steudel et al., 2024; Nordin et al., 2019), and electrocardiographic abnormalities such as QRS prolongation or bundle branch block (Parisi et al., 2023; El Sayed et al., 2023; Morimoto et al., 2021) are hallmark features of cardiac involvement and are closely associated with disease severity and adverse clinical outcomes (Vijapurapu et al., 2019b). Notably, in East Asian populations—and particularly in Taiwan—a substantial proportion of adult Fabry cardiomyopathy is attributable to the late-onset IVS4+919G>A (GLA) variant identified through newborn-screening programs, which is characterized by predominant and often progressive cardiac involvement with relatively attenuated extracardiac manifestations, thereby providing a clinically relevant model for examining long-term, domain-specific patterns of cardiac remodeling (Lin et al., 2009). Taken together, these observations indicate that Fabry cardiomyopathy is inherently a multi-domain cardiac disease, not one defined by a single structural or functional abnormality. This analytical framework, however, is not population-specific and may be applicable to Fabry cardiomyopathy across genotypes and geographic regions, consistent with contemporary national consensus recommendations for Fabry disease management (Hung et al., 2024).

Despite extensive characterization of individual cardiac manifestations, current research and clinical assessment strategies remain largely domain-specific. Most prior studies have focused on isolated structural, mechanical, or electrical parameters, typically represented by left ventricular hypertrophy or mass indices, myocardial deformation measures, or electrocardiographic conduction markers, respectively. These domain-specific measures have been shown in prior literature to correlate with disease severity and adverse clinical outcomes in Fabry cardiomyopathy, supporting their clinical relevance as surrogate markers (Pieroni et al., 2021; El Sayed et al., 2023; Chang et al., 2024). However, existing studies have predominantly relied on cross-sectional analyses or relatively short follow-up intervals (Nordin et al., 2019; Schiffmann et al., 2001), and have generally evaluated treatment response on the basis of average changes in single metrics. Such approach implicitly assumes that abnormalities across cardiac domains evolve in parallel over time. In routine clinical practice, this assumption is frequently violated, as patients commonly exhibit discordant longitudinal changes across domains. This discordance is further compounded when assessments rely on conventional cardiac measurements that are subject to intrinsic variability and operator dependence (Knackstedt et al., 2015; Costa et al., 2014). Accordingly, a key unmet need lies in analytical frameworks capable of capturing longitudinal, cross-domain remodeling patterns while preserving within-patient temporal trajectories. Whether Fabry cardiomyopathy progresses predominantly through synchronous remodeling across domains or through domain-specific, partially independent trajectories over time therefore remains incompletely understood. Cross-sectional studies have established LVH and increased LV mass as structural markers, impaired longitudinal strain as a mechanical marker, and QRS prolongation and conduction abnormalities as electrical markers, but they cannot determine whether these domains change synchronously within individual patients.

## Materials and methods

### Study design and population

This retrospective multicenter longitudinal study included 38 patients managed across three geographically distinct affiliated hospitals within the MacKay Memorial Hospital system, located in Taipei, Tamsui, and Hsinchu. Eligible patients had genetically and/or enzymatically confirmed Fabry disease, a baseline 12-lead ECG and transthoracic echocardiogram, and a follow-up ECG and echocardiogram. For patients who initiated ERT, baseline was the first eligible cardiac evaluation before or at treatment initiation; for untreated patients, baseline was the first eligible evaluation within the study period. Follow-up was the most recent eligible evaluation at least 6 months after baseline; all 38 patients met this minimum interval. Although no fixed research-visit schedule was imposed, ECG and echocardiographic reassessment was performed at least annually as part of routine longitudinal Fabry cardiac surveillance. No pre-specified exclusion window after hospitalization or an acute loading-condition change was applied. Treatment status reflected real-world clinical practice and was determined from medical records. The study protocol was approved by the institutional review board of MacKay Memorial Hospital, with a waiver of informed consent owing to the retrospective design.

### AI-based echocardiographic analysis

Echocardiographic quantification was performed using a vendor-independent, deep learning–based analysis platform developed by Us2.ai (Singapore), which has received United States Food and Drug Administration (FDA) 510(k) clearance for automated echocardiographic measurements in clinical use. This platform has been previously applied in randomized clinical echocardiographic studies for automated and standardized quantification of cardiac structure and function and was applied in the present study to standard transthoracic echocardiographic datasets (Sakamoto et al., 2026). Quantitative structural, mechanical, and diastolic parameters were derived in a fully automated manner, with the primary objective of improving measurement consistency and longitudinal reproducibility across serial examinations rather than generating diagnostic classifications. Left ventricular structural and systolic parameters, including left ventricular mass index (LVMi), left ventricular end-diastolic volume index (LVEDVi), and left ventricular ejection fraction (LVEF), were automatically quantified from standardized apical views using a biplane Simpson framework consistent with contemporary guideline recommendations (Lang et al., 2015). Myocardial mechanical function was assessed using AI-driven two-dimensional speckle-tracking deformation analysis of the left ventricle, with left ventricular global longitudinal strain (LVGLS) explicitly defined as the average peak systolic longitudinal strain of the left ventricular myocardium. LVGLS was treated as a continuous measure and processed using the same standardized AI-assisted workflow at baseline and follow-up. Left ventricular diastolic function was evaluated using continuous Doppler-based parameters. Tissue Doppler signals at the lateral mitral annulus were automatically identified to obtain early diastolic myocardial velocity (lateral *e*′) as an index of myocardial relaxation, and transmitral pulsed-wave Doppler data were integrated to derive early mitral inflow velocity (*E*), enabling calculation of the lateral *E*/*e*′ ratio as a noninvasive surrogate of left ventricular filling pressure. All echocardiographic measurements were obtained from standardized apical views with built-in quality control, and automated outputs underwent visual review to ensure anatomical plausibility and physiological coherence across the cardiac cycle. Source echocardiograms were acquired by trained sonographers at all three hospitals using standardized acquisition protocols and standard echocardiographic views and were stored as DICOM datasets. Examinations were performed on commercially available GE and Philips ultrasound systems. For retrospective analysis, identical view-selection, measurement definitions, and quality-control procedures were applied across hospitals and at both time points. The vendor-independent workflow classified two-dimensional videos and Doppler images to identify standard apical views, selected the loop with the highest view-classifier confidence, and performed automated cardiac-cycle selection, endocardial tracking, and strain estimation, followed by cardiologist review for anatomical plausibility (Myhre et al., 2024). The platform served as a standardized measurement pipeline rather than a diagnostic or prognostic classifier. Electrical–mechanical remodeling patterns were assigned outside the AI system using pre-specified, directly interpretable rules based on longitudinal changes in QRS duration and LVGLS. Each phenotype assignment was therefore directly traceable to the underlying clinical measurements. The pattern-recognition framework thus relied on transparent rule-based classification of standardized longitudinal measurements rather than on model-generated diagnostic, prognostic, or treatment predictions.

### Cardiac assessments and domain definition

Cardiac remodeling was evaluated across three predefined domains—structural, mechanical, and electrical—based on established pathophysiological frameworks describing myocardial involvement in Fabry cardiomyopathy and other infiltrative or hypertrophic cardiomyopathies, in which abnormalities in myocardial structure, contractile function, and electrical conduction have been shown to evolve in a partially dissociated manner (Pieroni et al., 2021; Nordin et al., 2019). Structural remodeling was assessed using left ventricular mass index, a widely accepted marker of myocardial hypertrophy and disease burden (Lang et al., 2015). Mechanical remodeling was evaluated using left ventricular global longitudinal strain, which reflects subclinical myocardial dysfunction and has been shown to detect early contractile impairment beyond conventional systolic indices (Vijapurapu et al., 2019a). Both structural and mechanical parameters were derived from echocardiographic assessments processed using the AI-based analysis platform described above. Electrical remodeling was evaluated using standard 12-lead electrocardiography (ECG), with QRS duration selected as an index of intraventricular conduction delay and myocardial involvement of the cardiac conduction system (Namdar et al., 2011). Standard 12-lead ECGs were obtained using Philips electrocardiograph systems. QRS duration was automatically computed by the device and reported digitally, then reviewed by a cardiologist against the tracing; no measurements were obtained from printed or scanned ECGs. Atrial fibrillation does not preclude QRS duration measurement, whereas bundle branch block and ventricular pacing may alter ventricular activation; QRS duration was therefore interpreted as a pragmatic marker of the observed ventricular electrical phenotype rather than of intrinsic conduction disease alone. All AI-derived echocardiographic outputs were reviewed and verified by experienced cardiologists prior to inclusion in the analysis. All measurements were obtained as part of routine clinical care, and identical acquisition protocols, parameter definitions, and analysis approaches were applied at baseline and follow-up to ensure longitudinal comparability.

### Longitudinal change and binary classification

Longitudinal change (Δ) for each parameter was defined as the follow-up value minus the corresponding baseline value at the individual patient level. The primary analytical focus was on within-patient change over time rather than cross-sectional between-patient comparisons. To facilitate cross-domain comparison, directional changes were further categorized using a binary framework. For each domain, changes were classified as adverse or non-adverse according to pre-specified criteria reflecting clinically relevant directionality (e.g., QRS prolongation for electrical remodeling, less-negative shift in LVGLS for mechanical remodeling and increase in LVMi for structural remodeling). Accordingly, this framework emphasizes sustained longitudinal directionality over multi-year follow-up, rather than minor fluctuations arising from physiological variation or measurement noise. The binary classification was applied solely as a conceptual tool to assess directional concordance or discordance across domains over time, and not to define biological disease subtypes. Continuous values were retained for descriptive analyses and graphical visualization.

### Electrical–mechanical remodeling phenotypes

Based on the direction of long-term within-patient changes in QRS duration and LVGLS between baseline and follow-up, patients were categorized into four predefined electrical–mechanical remodeling phenotypes. Directional change was assessed for each parameter independently, and classification was performed without applying predefined cutoff values or percentage thresholds. The four phenotypes were defined as follows: concordant favorable, characterized by non-adverse changes in both QRS duration and LVGLS; electrical-leading adverse, characterized by an adverse change in QRS duration with a non-adverse change in LVGLS; mechanical-leading adverse, characterized by a non-adverse change in QRS duration with an adverse change in LVGLS; and concordant adverse, characterized by adverse changes in both parameters. This directional framework allowed electrical and mechanical remodeling behaviors to be evaluated concurrently while preserving within-patient longitudinal trajectories. Structural remodeling, represented by LVMi, and treatment exposure were examined as contextual features within each electrical–mechanical phenotype rather than as independent classification axes. This approach was adopted to avoid over-fragmentation of phenotypic groups and to maintain interpretability of cross-domain remodeling patterns within a limited cohort size. The analytical workflow is summarized in Supplementary Figure S1.

### Statistical analysis

Continuous variables are presented as mean ± standard deviation, and categorical variables as counts and percentages. For longitudinal analysis, within-patient changes between baseline and follow-up were assessed using paired *t*-tests. Between-group comparisons according to treatment status were performed using independent-samples *t*-tests for continuous variables, with choice of parametric testing based on inspection of data distribution. Categorical variables were compared using the Chi-square test. Differences in the distribution of electrical–mechanical remodeling patterns between treated and untreated groups were evaluated using a Chi-square test applied to contingency tables. Given the exploratory, hypothesis-generating nature of this focused analysis, statistical interpretation emphasized descriptive longitudinal patterns and cross-domain behavior rather than causal inference. A two-sided *p*-value <0.05 was considered statistically significant. All analyses were performed using STATA, and figures were generated using Python. Annualized changes were calculated as follow-up minus baseline divided by follow-up duration in years. Because annualization can be unstable over short intervals, a sensitivity analysis was restricted to participants with at least 1 year of follow-up. Exploratory multivariable linear regression models evaluated baseline-to-follow-up changes in QRS duration, AI-assisted LVGLS, and LVMi after accounting for age at diagnosis, sex, follow-up duration, diabetes mellitus, hypertension, coronary artery disease, and chronic kidney disease (CKD) stage ≥3; heteroskedasticity-consistent (HC3) robust standard errors were used. The overall cohort comprised 38 patients.

## Results

### Baseline clinical characteristics of the study population

The study cohort consisted of 38 patients with Fabry disease who had paired baseline and follow-up cardiac assessments, enabling a longitudinal within-patient analysis. Patients were managed across three geographically distinct affiliated hospitals within the MacKay Memorial Hospital system, located in Taipei, Tamsui, and Hsinchu. Baseline characteristics are summarized in Table 1. The mean age at diagnosis was 63.7 ± 12.2 years, and 33 patients (86.8%) were male. The mean body mass index was 23.9 ± 3.9 kg/m^2^, and the mean baseline heart rate was 70.3 ± 8.8 beats per minute. The median follow-up duration was 5.3 years (interquartile range, 2.8–7.1 years). Cardiovascular comorbidities were frequently observed. Hypertension was present in 24 patients (63.2%), diabetes mellitus in 7 patients (18.4%), and documented coronary artery disease in 5 patients (13.2%). A history of heart failure was recorded in 10 patients (26.3%). Chronic kidney disease stage ≥3, defined as an estimated glomerular filtration rate <60 mL/min/1.73 m^2^, was present in 20 patients (52.6%). Prior ischemic stroke was documented in 7 patients (18.4%). The male predominance is consistent with the X-linked inheritance of Fabry disease: hemizygous men generally develop earlier and more clinically apparent manifestations than heterozygous women and are therefore more likely to be recognized and diagnosed, particularly in a cohort enriched for cardiac involvement. Age at diagnosis was 53.4 ± 8.0 years in classical Fabry disease and 66.1 ± 11.8 years in the cardiac variant.

**Table 1:** Baseline characteristics of the study cohort (*n* = 38).

Characteristic	Value
Age at diagnosis, years	63.7 ± 12.2
Classical Fabry disease, years	53.4 ± 8.0
Cardiac variant, years	66.1 ± 11.8
Male sex, *n* (%)	33 (86.8%)
Body mass index, kg/m^2^	23.9 ± 3.9
Baseline heart rate, bpm	70.3 ± 8.8
Hypertension, *n* (%)	24 (63.2%)
Diabetes mellitus, *n* (%)	7 (18.4%)
Coronary artery disease, *n* (%)	5 (13.2%)
Heart failure, *n* (%)	10 (26.3%)
CKD stage ≥3 (eGFR <60 mL/min/1.73 m^2^), *n* (%)	20 (52.6%)
Prior stroke, *n* (%)	7 (18.4%)
Atrial fibrillation, *n* (%)	9 (23.7%)
Supraventricular tachycardia, *n* (%)	1 (2.6%)
Ventricular tachycardia, *n* (%)	4 (10.5%)
AV block, *n* (%)	9 (23.7%)
Bundle branch block, *n* (%)	12 (31.6%)
Implanted cardiac rhythm device, n (%)	7 (18.4%)
Genotype	
Classical	7 (18.4%)
Cardiac variant	31 (81.6%)
ERT treatment, *n* (%)	22 (57.9%)

Values are presented as mean ± SD or *n* (%), as appropriate. CKD, chronic kidney disease; eGFR, estimated glomerular filtration rate; AV, atrioventricular; ERT, enzyme replacement therapy.

Baseline rhythm and conduction abnormalities were common. Atrial fibrillation was present in 9 patients (23.7%), supraventricular tachycardia in 1 patient (2.6%), and ventricular tachycardia in 4 patients (10.5%). Atrioventricular block was documented in 9 patients (23.7%), and bundle branch block in 12 patients (31.6%). Seven patients had an implanted cardiac rhythm device at baseline and follow-up: five dual-chamber permanent pacemakers and two implantable cardioverter-defibrillator (ICD) systems with pacing capability. No patient had cardiac resynchronization therapy (CRT) during the analyzed interval, and no documented interval device implantation, upgrade, pacing-programming change, cardiac surgery, or catheter ablation was identified. One pacemaker-to-CRT-P upgrade occurred in 2025 after the analyzed follow-up. Regarding genotype, 7 patients (18.4%) had classical Fabry disease, whereas 31 patients (81.6%) were classified as having the cardiac variant. ERT was administered in 22 patients (57.9%) during the observation period.

### Longitudinal within-patient changes across cardiac domains

Long-term within-patient changes in electrical, mechanical, and structural cardiac parameters are summarized in Table 2. Structural and mechanical parameters were derived from AI-assisted echocardiographic analysis using automated chamber quantification and speckle-tracking algorithms, while electrical parameters were obtained from standard 12-lead ECG. In the structural domain, interventricular septal thickness showed no significant change between baseline and follow-up (12.56 ± 3.61 mm vs. 12.91 ± 3.26 mm; Δ 0.35 mm, 95% CI − 0.91 to 1.62; *p* = 0.576). Left ventricular end-diastolic volume index (LVEDVi) decreased from 54.82 ± 23.40 to 49.95 ± 15.04 mL/m^2^ (Δ − 4.86 mL/m^2^, 95% CI − 12.90 to 3.18; *p* = 0.227). LVMi increased from 177.35 ± 74.48 to 181.67 ± 73.92 g/m^2^ (Δ 4.32 g/m^2^, 95% CI − 6.28 to 14.91; *p* = 0.415). When stratified by ERT status, the longitudinal change in LVMi differed significantly between groups, increasing in untreated patients and decreasing in ERT-treated patients (+23.24 ± 31.31 vs. −9.45 ± 25.68 g/m^2^; *p* = 0.001; Table 3).

**Table 2:** Long-term within-patient changes across electrical, mechanical, and structural cardiac domains.

Parameter	Baseline	Follow-up	Δ Mean difference (95% CI) Follow-up − baseline	*p*-value
Structural domain				
IVS, mm	12.56 ± 3.61	12.91 ± 3.26	0.35 (−0.91 to 1.62)	0.576
LVEDVi, mL/m^2^	54.82 ± 23.40	49.95 ± 15.04	−4.86 (−12.90 to 3.18)	0.227
LVMi, g/m^2^	177.35 ± 74.48	181.67 ± 73.92	4.32 (−6.28 to 14.91)	0.415
Mechanical domain				
LVEF, %	56.54 ± 11.47	61.52 ± 7.98	4.97 (0.82 to 9.13)	0.020
LVGLS, %	−14.74 ± 5.83	−16.69 ± 4.44	−1.95 (−4.05 to 0.14)	0.067
Lateral *e*′, cm/s	9.98 ± 4.76	9.63 ± 3.82	−0.35 (−2.12 to 1.42)	0.691
*E*/*e*′ ratio	8.50 ± 3.34	9.69 ± 5.52	1.19 (−0.98 to 3.35)	0.271
Maximal LAVi, mL/m^2^	27.00 ± 9.14	29.19 ± 12.16	2.19 (−4.84 to 9.22)	0.519
Electrical domain				
PR interval, ms	171.78 ± 31.01	181.36 ± 37.85	9.58 (0.90 to 18.26)	0.031
QRS duration, ms	128.21 ± 31.18	139.63 ± 37.95	11.42 (5.93 to 16.92)	<0.001
QTc, ms	464.24 ± 56.02	480.32 ± 51.21	16.08 (−1.87 to 34.04)	0.078

Values are presented as mean ± standard deviation. Long-term within-patient change (Δ) was defined as the difference between follow-up and baseline measurements (follow-up − baseline). Paired comparisons were performed using paired *t*-tests for continuous variables with available paired data. A two-sided *p*-value <0.05 was considered statistically significant. Negative values of left ventricular global longitudinal strain (LVGLS) indicate greater myocardial deformation. IVS, interventricular septal thickness; LVEDVi, left ventricular end-diastolic volume index; LVMi, left ventricular mass index; LAVi, left atrial volume index; QTc, heart rate–corrected QT interval.

**Table 3:** Long-term within-patient changes across cardiac domains stratified by ERT treatment status.

Variable	Untreated (*n* = 16) Δ (mean ± SD)	ERT-treated (*n* = 22) Δ (mean ± SD)	*p*-value
Structural domain			
Δ IVS thickness, mm	−0.52 ± 4.01	1.01 ± 3.37	0.229
Δ LVEDVi, mL/m^2^	−5.42 ± 18.72	−4.43 ± 26.47	0.903
Δ LV mass index, g/m^2^	23.24 ± 31.31	−9.45 ± 25.68	0.001
Mechanical domain			
Δ LVEF, %	0.93 ± 8.54	8.21 ± 13.98	0.077
Δ LVGLS, %	3.99 ± 5.01	−0.46 ± 3.61	0.003
Δ Lateral *e*′, cm/s	−0.74 ± 4.29	−0.10 ± 5.51	0.725
Δ Lateral *E*/*e*′	3.43 ± 5.49	−0.11 ± 5.71	0.109
Δ Maximal LAVi, mL/m^2^	−1.04 ± 8.86	3.95 ± 15.82	0.490
Electrical domain			
Δ PR interval, ms	11.20 ± 19.07	8.43 ± 29.90	0.754
Δ QRS duration, ms	11.56 ± 17.52	11.32 ± 16.52	0.965
Δ QTc interval, ms	22.53 ± 46.38	11.68 ± 59.05	0.555

Values are presented as mean ± standard deviation. Long-term within-patient change (Δ) was defined as the difference between follow-up and baseline measurements (follow-up − baseline). Comparisons between untreated and enzyme replacement therapy (ERT)–treated groups were performed using independent-sample *t*-tests or non-parametric equivalents, as appropriate. A two-sided *p*-value <0.05 was considered statistically significant. Negative values of global longitudinal strain (GLS) indicate greater myocardial deformation. IVS, interventricular septal thickness; LVEDVi, left ventricular end-diastolic volume index; LV mass index, left ventricular mass index; LVEF, left ventricular ejection fraction; GLS, global longitudinal strain; LAVi, left atrial volume index; PR, PR interval; QRS, QRS duration; QTc, heart rate–corrected QT interval.

In the mechanical domain, LVEF increased from 56.54 ± 11.47% at baseline to 61.52 ± 7.98% at follow-up (Δ 4.97, 95% CI 0.82 to 9.13; *p* = 0.020). AI-assisted LVGLS changed from −14.74 ± 5.83% to −16.69 ± 4.44% (Δ − 1.95 percentage points, 95% CI − 4.05 to 0.14; *p* = 0.067). No statistically significant longitudinal changes were observed in lateral *e*′ velocity, *E*/*e*′ ratio, or maximal left atrial volume index. In the electrical domain, PR interval increased from 171.78 ± 31.01 ms to 181.36 ± 37.85 ms (Δ 9.58 ms, 95% CI 0.90 to 18.26; *p* = 0.031). QRS duration increased from 128.21 ± 31.18 ms to 139.63 ± 37.95 ms (Δ 11.42 ms, 95% CI 5.93 to 16.92; *p* < 0.001). QTc interval increased from 464.24 ± 56.02 ms to 480.32 ± 51.21 ms, without reaching statistical significance (Δ 16.08 ms, 95% CI − 1.87 to 34.04; *p* = 0.078). Across all 38 patients, the annualized QRS change was 0.61 ± 11.60 ms/year (*p* = 0.746); the estimate was unstable over very short observation intervals. In the sensitivity analysis restricted to follow-up of at least 1 year, QRS duration increased by 2.34 ± 4.99 ms/year (95% CI, 0.65–4.03; *p* = 0.008), whereas annualized LVGLS and LVMi changes remained non-significant. QRS duration remained significantly increased after excluding patients with implanted cardiac rhythm devices (mean change, +9.48 ms; 95% CI, 3.38–15.59; *p* = 0.003) and after excluding those with bundle branch block and/or an implanted device at either time point (mean change, +5.63 ms; 95% CI, 1.36–9.90; *p* = 0.013). In exploratory multivariable analyses, no individual covariate was significantly associated with the magnitude of change in QRS duration, AI-assisted LVGLS, or LVMi.

### Electrical–mechanical remodeling phenotypes and structural burden

**Figure 1:**
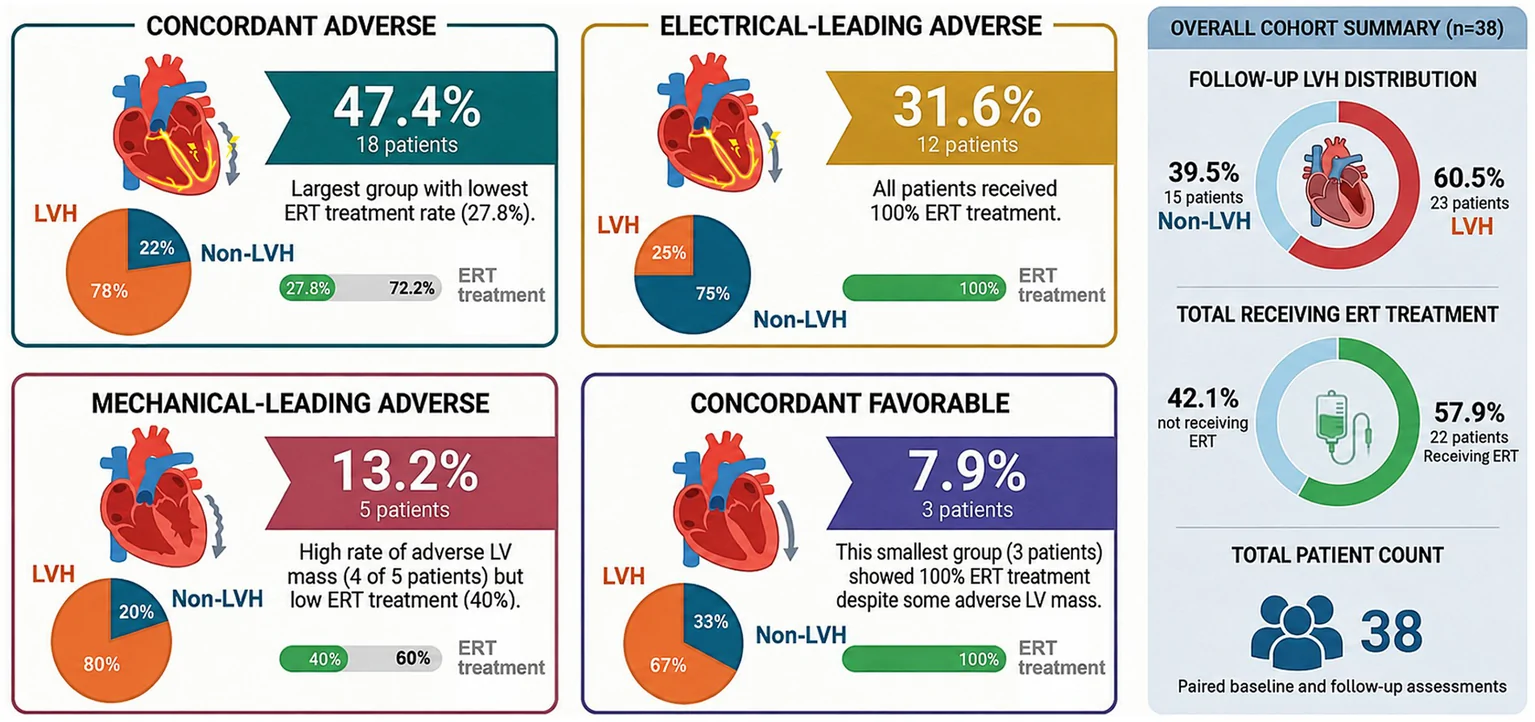
Mapping of longitudinal electrical–mechanical remodeling phenotypes and structural burden in Fabry cardiomyopathy. This figure summarizes the distribution of longitudinal electrical–mechanical remodeling phenotypes in 38 patients with paired LVGLS measurements, based on baseline-to-follow-up changes in QRS duration (electrical domain) and LVGLS (mechanical domain). Structural burden is shown as LVH status at follow-up and was not used as a third longitudinal classification axis. Patients were categorized as concordant adverse, electrical-leading adverse, mechanical-leading adverse, or concordant favorable. Within each phenotype, the distributions of LVH versus non-LVH status and ERT exposure are shown. Percentages are calculated relative to the study cohort or within each phenotype, as indicated. The figure illustrates heterogeneity across cardiac domains without implying a causal relationship between ERT and remodeling pattern.

Using directional changes in QRS duration and AI-assisted LVGLS, 38 patients were classified into four electrical–mechanical remodeling phenotypes (Table 4; Figure 1). Concordant adverse remodeling was identified in 18 patients (47.4%), electrical-leading adverse remodeling in 12 patients (31.6%), mechanical-leading adverse remodeling in 5 patients (13.2%), and concordant favorable remodeling in 3 patients (7.9%). At follow-up, LVH was present in 23 patients (60.5%) and absent in 15 patients (39.5%). Within the concordant adverse phenotype, 14 patients had LVH and 4 did not. In the electrical-leading adverse phenotype, LVH was present in 3 patients and absent in 9 patients. In the mechanical-leading adverse phenotype, LVH was present in 4 patients and absent in 1 patient. Among patients with concordant favorable remodeling, LVH was present in 2 patients and absent in 1 patient. All patients classified as concordant favorable (3 of 3, 100%) and electrical-leading adverse (12 of 12, 100%) received ERT; ERT was administered in 2 of 5 patients (40%) with mechanical-leading adverse remodeling and in 5 of 18 patients (27.8%) with concordant adverse remodeling (Table 4; Figure 1).

**Table 4:** Electrical–mechanical remodeling phenotypes with structural burden and ERT distribution (*n* = 38).

Electrical–mechanical phenotype	Definition (anchor markers)	Non-LVH, *n* (% total cohort)	LVH, *n* (% total cohort)	Total, *n* (% total cohort)	ERT treatment, *n* (% within phenotype)
Concordant favorable	Non-adverse QRS change + non-adverse LVGLS change	1 (2.6%)	2 (5.3%)	3 (7.9%)	3 (100%)
Electrical-leading adverse	Adverse QRS change + non-adverse LVGLS change	9 (23.7%)	3 (7.9%)	12 (31.6%)	12 (100%)
Mechanical-leading adverse	Non-adverse QRS change + adverse LVGLS change	1 (2.6%)	4 (10.5%)	5 (13.2%)	2 (40%)
Concordant adverse	Adverse QRS change + adverse LVGLS change	4 (10.5%)	14 (36.8%)	18 (47.4%)	5 (27.8%)
Total, *n* (% total cohort)		15 (39.5%)	23 (60.5%)	38 (100%)	22 (57.9%)

Values are presented as number of patients with percentages shown either relative to the total cohort or within each electrical–mechanical phenotype, as indicated. Electrical–mechanical remodeling phenotypes were defined according to the directional long-term within-patient changes in QRS duration and left ventricular global longitudinal strain (LVGLS). Adverse electrical change was defined as QRS prolongation, whereas adverse mechanical change was defined as a less-negative shift in LVGLS. Structural burden was summarized by the presence or absence of left ventricular hypertrophy (LVH) at follow-up. Enzyme replacement therapy (ERT) status reflects treatment exposure during the observation period. QRS, QRS duration; LVGLS, left ventricular global longitudinal strain; LVH, left ventricular hypertrophy.

### Longitudinal cross-domain remodeling patterns

**Figure 2:**
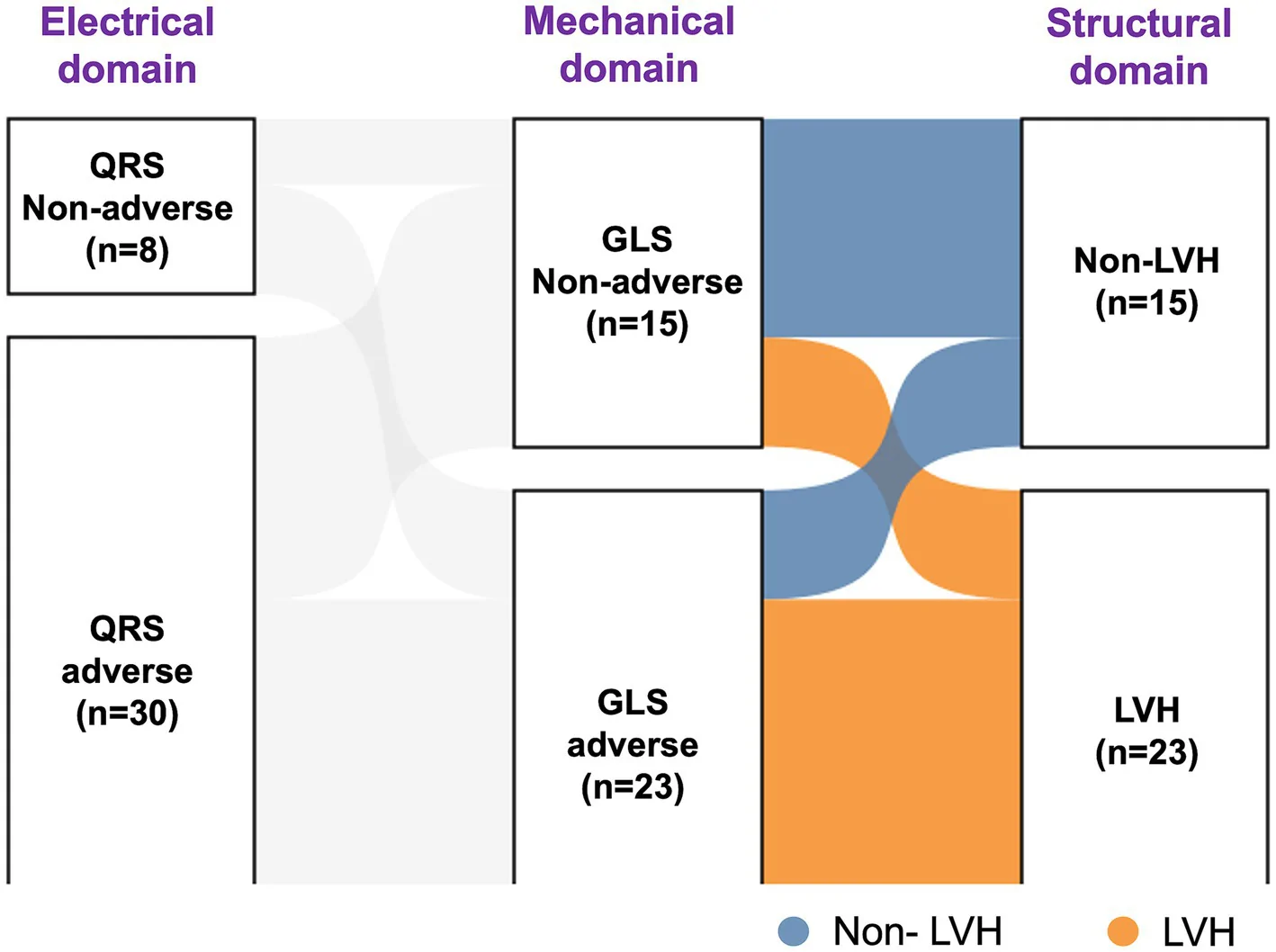
Directional alluvial map of electrical–mechanical remodeling patterns and follow-up structural burden in Fabry Cardiomyopathy. This directional alluvial map depicts long-term within-patient changes in the electrical and mechanical domains in 38 patients with paired LVGLS measurements, with structural burden overlaid as LVH status at follow-up. Electrical remodeling was defined by the direction of change in QRS duration, and mechanical remodeling by the direction of change in LVGLS. Structural status was not used as a longitudinal classification axis. The width of each flow corresponds to the number of patients in each pattern. The visualization demonstrates frequent dissociation between electrical and mechanical remodeling and their imperfect alignment with follow-up structural burden.

**Figure 3:**
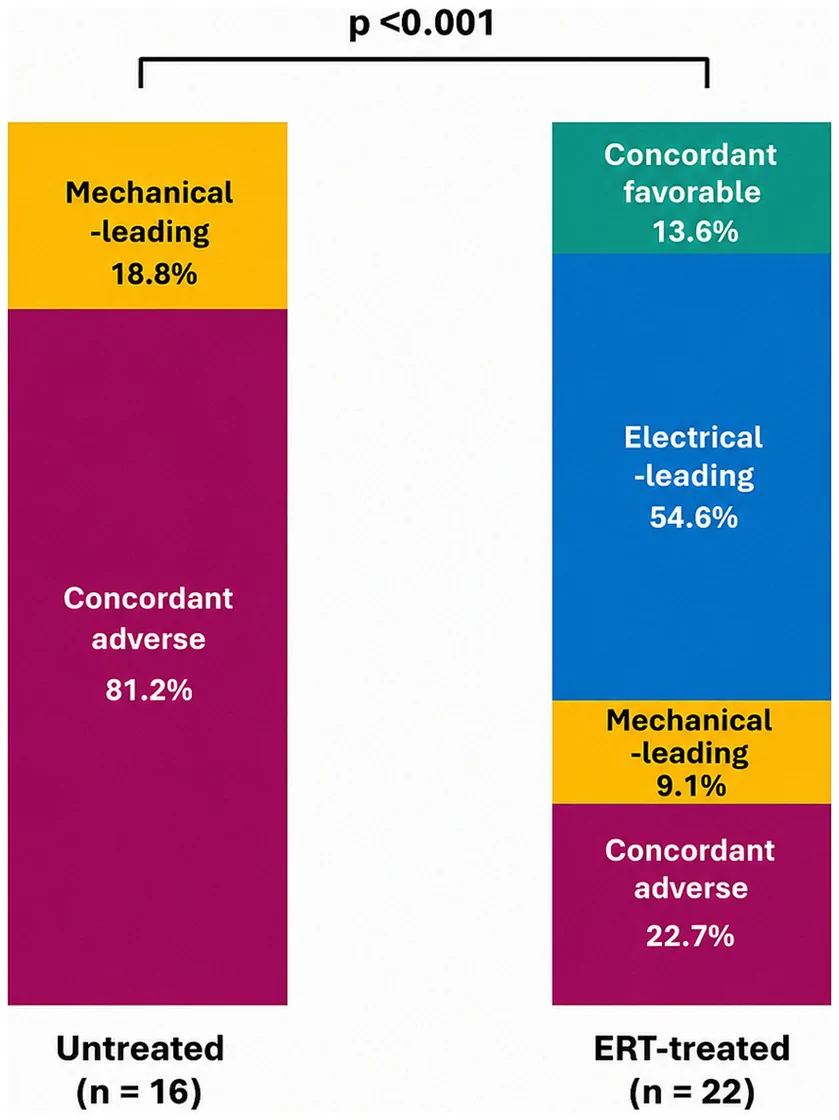
Electrical–mechanical remodeling patterns in untreated and ERT-treated patients. Bars depict the proportional composition of electrical–mechanical remodeling patterns within the untreated and ERT-treated groups (100% stacked). Patterns were defined from the direction of change in QRS duration and LVGLS. The overall distribution differed significantly between groups (Chi-square test, *p* < 0.001). *n* indicates the number of patients included in each group.

Structural remodeling was assessed longitudinally in the main analysis using baseline-to-follow-up changes in LVMi and other structural measures. In the phenotype visualization, however, the classification axes were longitudinal changes in QRS duration and LVGLS, while LVH was overlaid only as structural burden at follow-up. Figure 2 therefore depicts electrical–mechanical directional change patterns and their relationship to follow-up structural burden, without implying that LVH status itself represents a third longitudinal classification axis. Figure 3 illustrates the proportional composition of these patterns within untreated and ERT-treated groups. Untreated patients were characterized predominantly by concordant adverse remodeling (81.2%), whereas ERT-treated patients predominantly exhibited electrical-leading adverse remodeling (54.6%), with concordant favorable remodeling observed only in the treated group (13.6%). The overall phenotype distribution differed significantly between groups (Chi-square test, *p* < 0.001).

## Discussion

The central challenge in longitudinal follow-up of Fabry cardiomyopathy is less a shortage of diagnostic tools than a lack of concordance among cardiac parameters as the disease evolves over time. Electrical, mechanical, and structural abnormalities rarely emerge in parallel and often progress along divergent trajectories. When such discordant findings coexist during follow-up, their clinical significance is often difficult to interpret, and this remains an unresolved challenge in assessing disease progression. In the present study, we applied an AI-assisted functional processing framework to ensure that measurements obtained at different time points were evaluated under consistent analytical conditions. This approach was not intended to introduce new metrics, but to minimize operator-related variability and measurement drift. Within this standardized framework, asynchronous changes across cardiac domains were observed in most patients, including those receiving therapy, suggesting that non-parallel longitudinal trajectories are a defining feature of Fabry cardiomyopathy rather than incidental findings (Maurizi et al., 2024). These findings should be interpreted in the context of prior Fabry cardiac research. Most earlier studies have focused on individual cardiac domains—such as electrocardiographic abnormalities or selected imaging markers—and have relied predominantly on cross-sectional designs (Niemann et al., 2013). In parallel, pathological, imaging, and clinical investigations have demonstrated that distinct cardiac structures and functional systems become involved at different stages of disease progression, although these processes have typically been described in isolation (Azevedo et al., 2021; Weidemann et al., 2005). By aligning electrical, mechanical, and structural parameters along a shared longitudinal timeline, the present study allows domain-specific changes to be examined concurrently within individual patients, revealing patterns of dominance and divergence that are not apparent when each domain is considered separately. Adverse changes within individual cardiac domains are known to carry prognostic significance and to be associated with arrhythmic events and progression of heart failure (Chang et al., 2024; Namdar, 2016).

Adverse longitudinal consequences in Fabry cardiomyopathy are not confined to situations in which multiple cardiac domains deteriorate simultaneously; deterioration restricted to a single domain may still influence the overall disease trajectory. Longitudinal assessment therefore needs to consider not only the direction of change, but also which cardiac domain predominates over time. In the LVGLS analysis, concordant adverse remodeling was the most frequent phenotype (47.4%), followed by electrical-leading adverse remodeling (31.6%), mechanical-leading adverse remodeling (13.2%), and concordant favorable remodeling (7.9%). Structural burden at follow-up was present across all four phenotypes and was not treated as a third longitudinal classification axis. Both concordant and discordant patterns occurred in untreated and ERT-treated patients, highlighting substantial heterogeneity of remodeling responses. As illustrated in Figure 2, this non-convergent behavior supports the view that Fabry cardiomyopathy follows heterogeneous, non-linear remodeling pathways rather than progression along a single dominant axis.

At the population level, untreated patients were dominated by concordant adverse remodeling, whereas ERT-treated patients predominantly exhibited electrical-leading adverse remodeling (Figure 3), and the overall distribution differed significantly between groups (*p* < 0.001). This descriptive comparison should not be interpreted as a treatment effect. Domain-specific heterogeneity remains biologically plausible because glycosphingolipid accumulation and downstream injury vary across cardiomyocytes, the microcirculation, and interstitial structures (Azevedo et al., 2021; Augusto et al., 2021; Knott et al., 2019). Prior studies have reported that myocardial deformation and hypertrophy may stabilize or partially reverse after ERT in some patients, although responses vary with disease stage and myocardial substrate (Weidemann et al., 2003; Spinelli et al., 2020; Nordin et al., 2018; Weidemann et al., 2009). An electrical-leading pattern may arise when QRS prolongation persists while mechanical measures remain stable or improve, whereas a mechanical-leading pattern indicates relative predominance of deformation impairment during follow-up; neither pattern should be regarded as a distinct treatment-response subtype.

The coexistence of concordant and discordant remodeling patterns during longitudinal follow-up therefore appears to be an inherent feature of Fabry cardiomyopathy rather than an exception. In this setting, AI-assisted analysis is particularly relevant. Cardiac involvement in Fabry disease typically progresses slowly, with subtle changes and pronounced heterogeneity across domains, and longitudinal behavior is frequently asynchronous. Accurate interpretation thus depends critically on measurement consistency over time. Conventional manual or semi-automated workflows, however, are vulnerable to operator-dependent variability that may obscure small but clinically meaningful longitudinal changes. The AI-assisted functional processing framework used in the present study provides a stable and reproducible reference for serial assessment, preserving longitudinal signals across cardiac domains. Several limitations merit consideration. The modest sample size reflects the rarity of Fabry disease and limits formal prognostic inference. Moreover, the present study was designed to characterize longitudinal remodeling patterns rather than to define biological subtypes, and some subtle changes may not have been fully captured. Nonetheless, the within-patient longitudinal design and the use of a consistent analytical framework support the robustness of the observed findings. AI therefore functioned as the enabling measurement infrastructure rather than as an autonomous classifier: applying the same vendor-independent workflow at both time points reduced reader and post-processing variation and enabled longitudinal alignment of LVGLS and LVMi with QRS duration. This standardized workflow is particularly valuable for rare diseases such as Fabry cardiomyopathy, for which current international guidelines—including the Taiwan Society of Cardiology (TSOC) consensus—recommend close longitudinal monitoring to assess treatment efficacy and identify patients at risk of a suboptimal response, a practice that generates large serial imaging datasets that are difficult to quantify and compare using manual, reader-dependent methods (Hung et al., 2024). The clinical relevance of nonparallel remodeling lies in the limitations of single-domain surveillance. Stability in LVMi, LVGLS, or QRS duration alone may not indicate stability of the overall cardiac phenotype. Integrating serial ECG, deformation, and structural measurements may provide a more complete assessment and prompt review of conduction disease, pacing, loading conditions, treatment exposure, image quality, and reassessment timing. These phenotypes remain exploratory and should not yet determine treatment or individual risk stratification.

This study has several limitations. First, it was a retrospective multicenter study conducted across three geographically distinct affiliated hospitals within a single health system, with a small, predominantly male cohort. The modest sample size limits statistical power. Requiring paired ECG and echocardiographic examinations of adequate quality may also have introduced selection and survivor bias. Second, follow-up duration varied and only two assessment time points were available per participant; the temporal sequence, nonlinear evolution, and short-term variability of electrical, mechanical, and structural changes could not be characterized, and the annualized and ≥1-year sensitivity analyses cannot substitute for repeated intermediate measurements. No pre-specified maximum ECG–echocardiography pairing interval or exclusion window after hospitalization or an acute loading-condition change was applied, which may have introduced additional measurement-context variability. Third, QRS duration represents the observed ventricular electrical phenotype and may be influenced by bundle branch block and ventricular pacing. Sensitivity analyses excluding patients with an implanted cardiac rhythm device and/or bundle branch block supported the robustness of the overall QRS findings; however, quantitative ventricular pacing burden and detailed longitudinal device-interrogation data were unavailable, stable device status does not exclude an influence of pacing on QRS duration. Fourth, the AI-assisted echocardiographic workflow provided standardized processing across time points with cardiologist quality review, but retrospective image quality, loading conditions, and residual tracking or measurement variability may still have affected estimates of change. Fifth, ERT exposure was neither randomized nor standardized, so comparisons according to ERT status may be affected by confounding by indication, treatment duration, baseline disease severity, genotype, comorbidities, and concomitant therapy. Although exploratory multivariable models accounted for age at diagnosis, sex, follow-up duration, diabetes mellitus, hypertension, coronary artery disease, and CKD stage ≥3, the small cohort limited the number and complexity of covariates that could be examined, and residual and unmeasured confounding therefore remain; ERT-related comparisons are accordingly descriptive and should not be interpreted as causal treatment effects. Sixth, because the AI platform functioned as a measurement pipeline rather than a diagnostic or prognostic classifier, the remodeling-pattern assignment was performed outside the AI system using pre-specified, directly interpretable rules. Seventh, the directional electrical–mechanical phenotypes were exploratory and based on the direction of within-patient change; values close to zero may be susceptible to measurement variability, and the resulting categories should not be interpreted as validated biological or treatment-response subtypes. Given the two-time-point design and the pre-specified clinical objective of characterizing directional concordance and discordance, the interpretable directional framework was retained for the present analysis. Longitudinal clustering remains an important future extension for cohorts with denser serial measurements. Finally, the study did not evaluate adjudicated cardiovascular outcomes; therefore, the proposed phenotypes cannot currently be used to guide treatment, device implantation, or individual risk prediction. External validation in larger, sex-balanced, multicenter prospective cohorts with repeated assessments, detailed treatment and device data, and adjudicated clinical outcomes is required.

In summary, Fabry cardiomyopathy is characterized by frequent non-parallel longitudinal changes across electrical, mechanical, and structural cardiac domains. Concordant adverse remodeling was the most common electrical–mechanical pattern, but substantial heterogeneity was present across the cohort. Phenotype distributions differed significantly according to ERT exposure. Standardized multi-domain assessment may therefore provide a more complete account of disease evolution than surveillance based on any single cardiac parameter.

## Data Availability

The raw data supporting the conclusions of this article will be made available by the authors, without undue reservation.
