# Supplementary material for: AI-assisted longitudinal cardiac phenotyping identifies domain-specific remodeling patterns in Fabry cardiomyopathy
Source: Front Artif Intell. 2026 Sep 10;9:1800355. doi: 10.3389/frai.2026.1800355 (PMC13601274; doi:10.3389/frai.2026.1800355)
Supplement: Supplementary file 1 [file Supplementary_file_1.DOCX]

**Figure S1. Analytical framework for longitudinal electrical–mechanical phenotyping in Fabry cardiomyopathy**

Schematic overview of the analytical framework used for longitudinal electrical–mechanical phenotyping in Fabry cardiomyopathy. Paired baseline and follow-up electrocardiographic and echocardiographic data were processed using standardized retrospective procedures. Mechanical remodeling was assessed using change in left ventricular global longitudinal strain (LVGLS). Electrical remodeling was assessed using change in QRS duration. Within-patient changes were classified according to direction and integrated into four phenotypes: concordant adverse, electrical-leading adverse, mechanical-leading adverse, and concordant favorable. Phenotypes were contextualized according to follow-up structural burden (LVMi, wall thickness, and LVH status) and enzyme replacement therapy exposure. Δ indicates follow-up minus baseline. LVGLS, left ventricular global longitudinal strain; LVMi, left ventricular mass index; LVH, left ventricular hypertrophy; ECG, electrocardiography. The overall cohort comprised 38 patients.
